# Supplementary material for: Modeling the impact of public response on the COVID-19 pandemic in Ontario
Source: PLoS One. 2021 Apr 14;16(4):e0249456. doi: 10.1371/journal.pone.0249456 (PMC8046220; doi:10.1371/journal.pone.0249456)
Supplement: Attachment — Submitted filename: COVID___PLOS_One___Response_To_Reviewers.pdf [file pone.0249456.s001.pdf]

Abdallah M. Samy, PhD
Academic Editor
PLOS ONE

January 26, 2021

Re: Modeling the impact of public response on the COVID-19 pandemic in Ontario

Dear Editor,

We want to begin by thanking the editor and the reviewers for their careful consideration of our manuscript. We especially want to thank the reviewers for their assessment of our work and reviewer 1 for the many helpful comments. We have addressed the issues raised by reviewer 1 and summarize our response to revisions here.

In response to the first point, we have added extra language to the manuscript to clarify this distinction. In particular, we assume that anyone who is exposed enough as to be contagious will eventually become infectious. Individuals who are exposed to the virus but do not become contagious then remain in the S (susceptible) category. Implicitly we are assuming that their brief exposure was not enough to acquire antibodies, and so the influence of these individuals is still captured in the model as they still fill the susceptible pool. In regards to the reviewers second point, we calculated a mean infectious period of $1/(\alpha+\delta) \approx 18$ days. We have made this calculation more clear in the manuscript.

In response to the third and fourth points, we thank the reviewer for the suggestion and agree that it would be an interesting avenue for future investigation. Including the effect of hospitalization into our model is beyond the scope of the current manuscript, but will be considered in a future work. In response to the fifth point, we have made the change in Figure 5. For the sixth point, we have included extra language in the manuscript detailing that while public behaviour changed, official public health policy was constant. This suggests that some other process was behind the shift in behaviour observed.

Finally, for the reviewers seventh point we are grateful for the suggestion. Certainly comorbid infectious diseases that spread via similar mechanisms would be interesting to investigate in general. Such a project would prove to be an enlightening usage of the model we present in this project. However, for the current year influenza rates have fallen significantly (likely as a result of the enhanced public health measures) so the influence of this virus on the spread of SARS-CoV-2 may not be as relevant in our current study.

Sincerely,

Brydon Eastman, PhD Candidate,
Department of Applied Mathematics,
University of Waterloo
Waterloo, Ontario, Canada
b2eastma@uwaterloo.ca
